# Detecting congenital malformations - Lessons learned from the Mpepu study, Botswana

**DOI:** 10.1371/journal.pone.0173800

**Published:** 2017-03-24

**Authors:** Gbolahan Ajibola, Rebecca Zash, Roger L. Shapiro, Oganne Batlang, Kerapetse Botebele, Kara Bennett, Florence Chilisa, Erik von Widenfelt, Joseph Makhema, Shahin Lockman, Lewis B. Holmes, Kathleen M Powis

**Affiliations:** 1 Botswana Harvard AIDS Institute Partnership, Gaborone, Botswana; 2 Division of Infectious Diseases, Beth Israel Deaconess Medical Center, Boston, Massachusetts, United States of America; 3 Department of Immunology and Infectious Diseases, Harvard T.H. Chan School of Public Health, Boston, Massachusetts, United States of America; 4 Bennett Statistical Consulting, Inc., Ballston Lake, New York, United States of America; 5 Infectious Disease Unit, Brigham and Women's Hospital, Boston, Massachusetts, United States of America; 6 Genetics Unit, MassGeneral Hospital for Children, Boston, Massachusetts, United States of America; 7 Department of Internal Medicine, Massachusetts General Hospital, Boston, Massachusetts, United States of America; 8 Global Health, MassGeneral Hospital for Children, Boston, Massachusetts, United States of America; Azienda Ospedaliera Universitaria di Perugia, ITALY

## Abstract

**Introduction:**

A large and increasing number of HIV-infected women are conceiving on antiretroviral treatment (ART). While most antiretrovirals are considered safe in pregnancy, monitoring for rare pregnancy and infant adverse outcomes is warranted.

**Methods:**

We conducted a retrospective secondary analysis nested within a clinical trial of infant cotrimoxazole vs. placebo prophylaxis in Botswana (the Mpepu Study). Infants were examined at birth, and at least every 3 months through 18 months of age. Abnormal physical findings and diagnostic testing revealing malformations were documented. Post hoc, a geneticist classified all reported malformations based on available documentation. Structural malformations with surgical, medical or cosmetic importance were classified as major malformations. We present a descriptive analysis of identified malformations.

**Results:**

Between 2011 and 2014, 2,933 HIV-infected women who enrolled in the Mpepu study delivered 2,971 live-born infants. Study staff conducted 2,944 (99%) newborn exams. One thousand eighty-eight (38%) women were taking ART at conception; 1,147 (40%) started ART during pregnancy; 442 (15%) received zidovudine monotherapy; and 223 (7%) received no antiretroviral during pregnancy. Of 33 reported anomalies, 25 (76%) met congenital malformations criteria, 10 (30%) were classified as major malformations, 4 (40%) of which were identified after the birth exam.

**Discussion:**

Our results highlight the importance of staff training on identification of congenital malformations, programmatic monitoring beyond the birth examination and the value of geneticist involvement in the malformations classification process in resource-limited settings. These elements will be important to fully define antiretroviral drug safety in pregnancy.

**Significance:**

Surveillance systems for monitoring the safety of antiretroviral use during pregnancy among HIV-infected women in resource-limited setting are lacking. The World Health Organization’s published programmatic recommendations for such surveillance systems represents the gold standard. We employed data from a clinical trial in Botswana, a country with a generalized HIV epidemic and high antiretroviral uptake by HIV-infected women, to highlight practical opportunities to strengthen congenital malformation surveillance systems in these settings where over 1 million HIV infected pregnant women reside.

**Trial registration:**

Clinical Trials.gov NCT01229761

## Introduction

The World Health Organization (WHO) recommends that all HIV-infected pregnant women receive 3-drug antiretroviral treatment (ART) in pregnancy and that ART initiated in pregnancy be continued for the woman’s lifetime [1]. ART use in pregnancy and during the breastfeeding period has been shown to significantly reduce the incidence of mother-to-child HIV transmission [2, 3, 4, 5]. With an estimated 1.5 million HIV-infected pregnant women eligible for ART [6], WHO has recognized that key research gaps exist with respect to short- and long-term effects of antiretroviral use in pregnancy, particularly first trimester exposure, and has called for the implementation of surveillance systems to ensure that *in utero* fetal antiretroviral exposure is not associated with congenital malformations [1]. Sites with generalized HIV-epidemics could contribute significant quantities of data to ART safety surveillance systems. However, these sites are predominately located in resource-limited settings, where few countries have surveillance programs in place, and those with a program are addressing challenges such as lack of trained health personnel to properly identify and classify the anomaly, lack of systematic and harmonized data collection processes, lack of allocation of appropriate health resources to surveillance programming, the need to establish a longitudinal exam and reporting component, and the lack of sufficient, and in some settings, any geneticists or a multidisciplinary team to validate findings and assist with definitive interventions and/or support care [7, 8].

We present descriptive findings and programmatic opportunities experienced in conducting a secondary retrospective analysis of congenital malformations occurring among HIV- and ART-exposed infants participating in the Mpepu study, in Botswana. The Mpepu study was designed to determine if extended use of infant cotrimoxazole versus placebo initiated between 14–34 days of life and continued through 15 months offered a survival benefit to HIV-exposed uninfected infants. Lessons from our findings can inform planned ART safety surveillance systems in similarly resourced settings where generalized HIV-epidemics exist.

## Methods

### Study population and monitoring

We performed a retrospective analysis of data from mother-infant pairs enrolled in the Mpepu study in Botswana between May 2011 and December 2014. The Mpepu study is a double-blinded randomized controlled trial designed to assess the efficacy and safety of infant cotrimoxazole vs. placebo prophylaxis taken from 14 days through 15 months of life by HIV-exposed uninfected (HEU) infants. Women were eligible to enroll in the study if they had documentation of their HIV-infected status, were citizens of Botswana, were 18 years-of-age or older, were between 26 weeks’ gestation and 34 days postpartum, had ability to provide informed written consent, and were willing to remain in the study area and attend study visits until the final visit at 18 months postpartum, with their infants. Mpepu study enrollment exclusion criteria prevented enrollment of infants with serious, life-threatening illnesses or congenital malformations that might have precluded survival to at least 18 months of life.

The Botswana Health Research Development Committee and the Office of Human Research Administration at Harvard T. H. Chan School of Public Health approved the Mpepu study. Women signed written informed consent to authorize study participation for themselves and their infants.

For the retrospective analysis of congenital malformations, mother-infant pairs were eligible for inclusion if there was documentation of a birth exam. At study enrollment, women provided verbal report of prior antiretroviral use, including date of initiation, regimens and self-reported adherence during pregnancy. Antiretroviral use was also abstracted from the women’s medical records, when possible, to validate the reported information.

Infant study visits were scheduled at birth, between the infant’s 14^th^ and 34^th^ day of life for randomization to Cotrimoxazole or placebo, and at 2, 3, 6, 9, 12, 15, and 18 months-of-life. Due to the fact that mother-infant pairs could enroll as late as 34 days after the birth of the infant, the first infant exam by a study clinician qualified as the birth exam, even if it occurred more than 72 hours after birth. Infants were examined by Mpepu study clinicians, including both nurses and physicians, at every scheduled study visit. The results of that exam were documented in an electronic data management system, in which full exam findings were required by organ system. At the onset of the Mpepu study, staff received training and demonstrated competency in conducting a newborn exam, as well as age appropriate exams through 18 months-of-life. During each study visit, the mother provided verbal report of any healthcare interactions since the last study visit and the child’s national health booklet was reviewed for further evidence of the need for medical interventions since the last visit. This information was recorded in the study’s electronic data management system and the external records were photocopied and retrained as study source documents.

We reviewed the study’s electronic data management system for reports of malformations documented during any study visit. Identification of either abnormal physical findings or presence of a congenital malformation resulted in a review of an infant’s entire study records, including source documentation of clinical notes from government health facilities where the infant may have been evaluated and managed between Mpepu study visits. Using source documentation, the timing of identification of congenital malformations, as well as presenting symptoms associated with anomalies were ascertained. In selected cases where the final intervention was not fully documented in Mpepu study records, caregivers were contacted directly and asked to explain the interventions. All documentation related to congenital malformations was reviewed by a Geneticist at MassGeneral Hospital *for* Children, who made a final decision on whether the documented aberrancy qualified as a congenital malformation. If deemed to be a congenital malformation, the Geneticist assigned a severity rating of either a major or minor congenital malformation, solely based upon source documentation without the use of photographs. In order for the finding to meet criteria for a major congenital malformation, it had to represent a structural abnormality with surgical, medical or cosmetic importance. A classification of minor malformation was assigned to those structural abnormalities that were not associated with need for surgical or medical interventions and had no cosmetic importance.

### Statistical methods

SAS, version 9.3 (SAS Institute, Cary, North Carolina, USA) was used to perform all statistical analyses. Mother and infant characteristics were evaluated, with means calculated on continuous variables with normal distributions, medians calculated for non-normally distributed continuous variables, and proportions calculated for categorical variables. Descriptive statistics were used to summarize all infant congenital malformations and *in utero* ARV exposures. No attempt was made to calculate an overall congenital malformation rate or compare the rate by timing of *in utero* antiretroviral exposure due to the bias inherent in the Mpepu protocol, with exclusion criteria that precluded the enrollment of infants with serious life-threatening illnesses or congenital malformations that might have prevented survival to at least 18 months of life. While these exclusion criteria were appropriate for the parent study, which was specifically designed to investigate survival to 18 months of life, it likely resulted in underreporting of congenital malformations. Furthermore, the retrospective nature of this analysis may also contribute to underreporting.

## Results

### Maternal—Infant baseline characteristics

**Fig 1:**
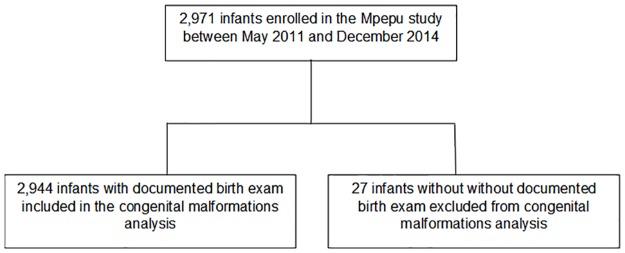
Study accrual flow diagram.

Between May 2011 and December 2014, 2,933 HIV-infected women and their 2,971 infants were enrolled in the Mpepu study. Almost all (n = 2,944, 99%) infants had documentation of a birth exam performed by a study clinician (Fig 1). Among the 2,906 women whose infants had birth exams, only 509 (18%) were enrolled during the antenatal period, while 2,397 (82%) were enrolled between delivery and 34 days postpartum. Characteristics of these mother-infant pairs are presented in Table 1. The mean age of women at delivery was 31.8 years [95% Confidence Interval (CI) 27.3–36.2] and the majority of women were multiparous, with only 14.4% of the women being nulliparous. Over 90% of women received at least one antiretroviral during pregnancy, with nearly 40% reporting use of 3-drug ART at time of conception. The majority of women gave birth to full-term infants with median gestational age at birth of 39 weeks [Interquartile Range (IQR) 37–40 weeks].

**Table 1:** Maternal-Infant characteristics.

**Maternal Characteristics**^a^ **(n = 2,906)**	
**Median maternal age (years) [IQR]**	31.8 [27.3–36.2]
**Gravida Including Current Pregnancy (#, %)**	
** 1**	419 (14.4%)
** 2**	686 (23.6%)
** 3**	757 (26.1%)
** 4 or more**	1044 (35.9%)
**Type of Pregnancy**	
** Singleton**	2862
** Twin**^b^	43
**Delivery Median CD4+ count (cells/μl) [IQR]**	500 [353–672]
**Antiretroviral Timing/Type**^c^ **(#, %)**	
** Triple Antiretroviral initiated before conception**	1139 (39.2%)
** Triple Antiretroviral initiated in 1^st^ trimester**	87 (3.0%)
** Triple Antiretroviral initiated in the 2^nd^/3^rd^ trimester**	1004 (34.5%)
** AZT monotherapy initiated in pregnancy**	488 (16.8%)
** No ANTIRETROVIRAL use in pregnancy**	188 (6.5%)
**Enrollment Site (#,%)**	
** Molepolole (Village)**	960 (33.0%)
** Lobatse (Town)**	237 (8.2%)
** Gaborone (City)**	1709 (58.8%)
**Marital Status (#, %)**	
** Single**	2357 (81.1%)
** Married/Cohabitating**	527 (18.1%)
** Widowed/Divorced/Other**	22 (0.8%)
**Education (#, %)**	
** None or Primary**	461 (15.8%)
** Secondary**	2225 (76.6%)
** University**	220 (7.6%)
**Electricity present in home (#, %)**	1544 (53.1%)
**Infant Characteristics**^d^ **(n = 2,944)**	
**Infant Sex (#, %)**	
** Male**	1449 (49.2%)
** Female**	1495 (50.8%)
**Median Gestational Age at Delivery [IQR]**	39 [37, 40]
**Median Anthropometric Measures**	
** Birth weight (kg) [IQR]**	
** Male Infants**	3.00 [2.64–3.30]
** Female Infants**	2.88 [2.59–3.20]
** Medeian Length (cm) [IQR]**	
** Male Infants**	50.0 [48.0–52.0]
** Female Infants**	50.0 [48.0–51.0]

^a^ Mothers for whom we had a documented infant birth exam

^b^ Of the 43 women delivering twins, 38 women enrolled both twins in the study while 5 women only enrolled one twin.

^c^ 49 women had documentation of triple antiretroviral use prior to delivery. However, timing of initiation was unknown

^d^ Infants with documented birth exam

### Congenital malformations

In total, 33 (1.1%) of the 2,944 infants had documentation of a malformation, 27 (0.9%) malformations identified by study clinicians and an additional 6 (0. 2%) abstracted from infant medical records following diagnostic tests or imaging leading to the identification of internal congenital malformations, (Table 2). Post-hoc review of reported malformations by a geneticist (LH) resulted in a total of 25 (76%) cases meeting criteria for congenital malformations, 10 (30%) classified as major congenital malformations. Four (40%) of the ten major malformations were first identified after the birth exam. Fifteen (45%) of the congenital malformations were classified as minor, 1 (0.07%) of which was identified 60 days after birth. Six did not qualify as a congenital malformation and there were insufficient details about 2 reported malformation cases to assign proper classification.

**Table 2:** Abnormal infant findings, timing of identification and subsequent congenital malformations classification.

Description of physical findings or Diagnostic test	# of Cases	Malformations Classification^a^^1^	Timing of Identification
**Ambiguous Genitalia**	1	Insufficient Details for Classification	Birth
**Anovesicular Fistula**^b^	1	Major Malformation	42 Days
**Biliary Atresia**^b^	2	Major Malformation	38 Days, 48 Days
**Congenital lymphedema**	1	Insufficient Details for Classification	15 Days
**Hyperextended Leg**	2	Non-Malformation	Birth (x2)
**Hypospadia**	1	Major Malformation	Birth
	1	Minor Malformation	Birth
**Jejunal Atresia**^b^	1	Major Malformation	5 Days
**Lower Limb Agenesis**	1	Major Malformation	Birth
**Missing Right Thumb**	1	Major Malformation	Birth
**Neonatal Teeth**	3	Minor Malformation	Birth (x3)
**Oral Cysts**	2	Non-Malformation	Birth (x2)
**Preauricular Skin Tag**	1	Minor Malformation	Birth
**Postaxial Polydactyly Type B**	9	Minor Malformation	Birth (x9)
**Talipes Equinus**	1	Minor Malformation	60 Days
**Talipes Equino- varus/valgus**	3	Major Malformation	Birth (x3)
**Trisomy 21**	1	Non-Malformation	Birth
**Umbilical Hernia**	1	Non-Malformation	Birth

^a^ Classification of congenital malformation was performed by a Geneticist blinded to maternal use of Antiretroviral in pregnancy, with classification of major malformation being assigned to reports of a structural abnormality with surgical, medical or cosmetic importance. A classification of minor malformation was assigned to reports of structural abnormalities that were not associated with a need for surgical or medical interventions and had no cosmetic importance.

^b^ Identified after the initial birth exam usually in the course of routine follow-up physical exams. All such findings were confirmed either with appropriate diagnostic testing (X-RAYS, CT-SCAN, MRI) or surgery where applicable.

Five of the ten infants experiencing major congenital malformations were exposed to triple antiretrovirals (ARVs) from conception, while three infants were not exposed to triple ARVs until the 2nd or 3rd trimester and two women who gave birth to infants with major malformations took zidovudine monotherapy initiated in the 3rd trimester. Among infants with malformations categorized as minor, ten were exposed to triple ARVs from conception, one from the second trimester and two from the third trimester, while the remaining two infants with minor malformations were exposed to ZDV-monotherapy initiated in the 2nd and 3rd trimester respectively.

## Discussion

Using data from a clinical trial in Botswana, we found that 40% of major congenital malformations were not visibly detectable during the birth exam but were identified during longitudinal follow-up. In resource-restricted settings, such as Botswana, the site of the Mpepu study, surveillance systems, if present, are often limited to the initial birth exam. Based upon our findings, such a system would understate the presence of major congenital malformations. Our findings underscore the importance of a longitudinal component in congenital malformations surveillance systems.

In our cohort, consisting of only live born infants with study enrollment criteria that precluded enrollment of infants unlikely to survive, including those with life threatening congenital malformations, the low number of congenital malformations observed was expected and represents a limitation of our retrospective secondary analysis. However, 18% of reported abnormalities did not meet the criteria for congenital malformations, employing a standard definition as adjudicated by a Geneticist. We may have been able to lower this misclassification rate through pre-specification of malformations of interest, as advocated by the WHO, the Centers for Disease Control and Prevention and ICBDSR in their general guidelines for surveillance programs for all birth defects [9]. However, discrepancies between reported and actual congenital malformations are not unique to resource-limited settings. Holmes and Westgate [10] reviewed physical findings documented by Pediatricians performing newborn exams from 1,000 consecutive deliveries involving live born infants, stillborn infants, and elective terminations of fetal malformations at a Boston-based hospital, and found 240 of the infants had documentation of an abnormal physical finding. Yet only 18 (1.8%) of the reported abnormalities actually represented major congenital malformations, using identical criteria to that used in this sub-study. Furthermore, the WHO Steering Group on antiretroviral Toxicity Surveillance [1], a panel of international experts and representatives of research agencies, would be well positioned to establish the major congenital malformations that are of concern.

Programmatic pre-specification of congenital malformations of concern would have provided the foundation from which specific training materials, including materials comparable to the atlas of selected congenital malformations published by WHO [11] could have been employed by our team. Ideally such materials could be centrally developed and distributed to resource-limited settings. Use of common training materials and course certification criteria, whether used by clinicians, allied health professionals, or in settings where health care providers represent a scare commodity, lay persons, would promote consistency in reporting across sites. Electronic medium-based training materials and proficiency exams would facilitate user access in settings where training experts may be limited, and pre-specification of congenital malformations would lend itself to creation of physical exam worksheets either available in electronic or paper format. These surveillance program features are likely to promote consistency in examinations and collection of data within and between sites and countries.

Reported malformations from the Mpepu study were classified by a Geneticist. This facilitated proper and consistent classification. Recognizing that Geneticists may not be available in resource-limited settings experiencing generalized HIV-epidemics argues for organizations such as WHO to create or support the creation of expert regional panels to promote proper classification of malformations, encourage case management and build the capacity of antiretroviral birth surveillance programs. Even in resource-limited settings, technology is progressing at a rate to support electronic (eHealth) and mobile health (mHealth) applications, providing for technologically viable mechanisms for medical experts to provide guidance or evaluation remotely [12–14].

In the Mpepu study, the robustness of our open-source data collection system allowed for capture of maternal antiretroviral use with initiation and stop dates, as well as infant exam documentation. Programmed embedded logic in our system ensured that clinicians completed all required forms and data at the time of the visit and that illogical data was corrected at the time an attempt was made to save a form electronically (e.g. entry of an antiretroviral discontinuation date before the date of initiation). Data was collected from three sites but stored centrally with de-identified data accessible to remotely located authorized users. Use of eHealth or mHealth applications could provide similar approaches, permitting either for real-time data entry or subsequent entry of data captured on paper-based forms from remote sites with centralized data storage and accessibility to authorized users. While confidentiality, security, and country contextual need to obtain written informed consent from parents of infants evaluated within such a program may require system customization at the country level [9, 15, 16], development of an electronic application with expert oversight creates the infrastructure for uniform collection and diagnosis of congenital malformations beyond a country level.

Congenital malformations are rare events. Pooling data collected through similar methodologies from multiple countries in sub Saharan Africa, where over 90% of HIV-infected pregnant women reside [17], would allow for more timely evaluation of the overall safety of ART in pregnancy. Currently, in sub-Saharan Africa, Botswana, South Africa, Malawi and Uganda [18, 19] are the first countries to pilot congenital anomalies monitoring programs in relation to the safety of antiretroviral drug use in pregnancy, including the occurrence of congenital malformations. We believe that these pilots will identify many of the same challenges identified in our retrospective analysis, but are better positioned to more accurately report on congenital anomaly rates.

The study teams in Botswana, Malawi and Uganda hope to pool data, and this represents a good starting point.

The virtual elimination of mother-to-child HIV-transmission with maternal use of ART in pregnancy represents one of the greatest global public health policy successes. However, the teratogenicity of these potent medications has not been fully exonerated. Establishing viable safety surveillance systems in resource-limited sites where the vast majority of ART is being used in pregnancy represents an urgent public health priority.
